# *Calendula officinalis* stimulate proliferation of mouse embryonic fibroblasts via expression of growth factors TGFβ1 and bFGF

**DOI:** 10.1186/s41232-019-0097-x

**Published:** 2019-04-20

**Authors:** Maryam Hormozi, Mohammadreza Gholami, Ayda Babaniazi, Anneh Mohammad Gharravi

**Affiliations:** 1 Razi Herbal Medicine Research Center, Lorestan University of Medical Sciences, Khorramabad, Iran; 2 Department of Biochemistry, Lorestan University of Medical Science, Khorramabad, Iran; 3 Department of Anatomical Sciences, Kermanshah University of Medical Sciences, Kermanshah, Iran; 4 Student Research Committee, Lorestan University of Medical Sciences, Khorramabad, Iran; 5 Tissue Engineering and Stem Cells Research Center, Shahroud University of Medical Sciences, Shahroud, Iran

**Keywords:** *Calendula officinalis*, bFGF, TGFβ1, Mouse embryonic fibroblasts

## Abstract

**Background:**

TGF-β has an important role in the process of wound healing and scar formation. The aim of this study is to determine the effects of ethanolic and methanolic extracts of *Calendula officinalis* on the expression of TGFβ1 and bFGF in the mouse embryonic fibroblast cells (MEFs).

**Methods:**

*Calendula officinalis* extract was purchased and different substances defined with gas chromatography and mass spectrometry. MEFs were prepared and after incubating for 15 min, cell viability analyzed. TGF β 1 and bFGF gene expression was evaluated by real-time PCR. TGFβ1 and bFGF protein expression analyzed by ELISA. The statistical analysis of data was done by using SPSS software. Differences were considered significant at (*P* < 0.05).

**Results:**

The results of the MTT test showed that the concentrations of 5 μg/ml and10 μg/ml were more suitable for cell proliferation. There was an increase in TGF β 1 gene expression in the MEFs. Expression of TGF β 1 gene remains the same after 24 h. Gene expression of bFGF showed a similar pattern with *TGF* β *1* expression for both solvents. Analysis of TGFβ1 protein expression showed an increase in TGFβ1 gene expression in the MEFs. Protein expression of bFGF in the MEFs increased at different concentrations at 12 and 24 h after treatment (*P* < 0.05 and *P* < 0.01 respectively).

**Conclusion:**

*Calendula officinalis* stimulates proliferation of MEFs. Calendula via increased expression of growth factors (TGFβ1 and bFGF) at the first 12 h and a decrease of these factors at 24 h after treatment may ameliorate function of the MEFs in the during wound healing.

## Background

Growth of new tissue and scar formation are two important issues in tissue injury. Fibroblasts are the main cells in the wound healing process because they migrate and proliferate to the injury site after 2 or 3 days. Then, the cells produce extracellular matrix especially collagen. Growth factors play important roles in the proliferation, migration, and production of ECM substances by fibroblasts. Recently, researches showed that the growth factor TGF-β has an important role in the process not only in wound healing but also in scar formation. TGF-β applies their effects via the intracellular SMAD pathway. [1, 2]. It has been shown that TGFβ1 activates angiogenesis by stimulating vascular smooth muscle cell migration [3–5]. The importance of this factor in creating and maintaining the vascular system has been proved by numerous studies. TGFβ1 and its receptors lead to fetal death due to impaired vessel formation [6, 7]. TGFβ1 induces the maturation of retinoic acid-inducible gene I (RIGI), therefore, stimulates the formation and intensification of interaction between epithelial cells and the basement membrane of the mural cells [8–10]. Also bFGF, (FGF 2 or FGF-β) contribute to control of endothelial cells and fibroblasts migration, which are responsible for angiogenesis and collagen formation of the epithelial layer [1, 9, 11].

*Calendula officinalis* L. (Asteraceae) has been traditionally used in the treatment of various diseases such as skin tumors, dermatological lesions, and swellings. Despite a long tradition of use of *Calendula officinalis* L., the biological aspect of its activity has not been explored properly. Recent research supports the medicinal potential of *Calendula officinalis* L. *Calendula officinalis* L. properties need to be investigated to determine their varied biological activities and mechanism of actions. To determine the effectiveness of the *Calendula officinalis* L. in the treatment of dermatologic disorders, more research is necessary to understand the action mechanism of this plant. Therefore, the aim of this study is to determine the effect of the *Calendula officinalis* L. extract on proliferation and expression of two important growth factors, TGFβ1 and bFGF, at the MEFs.

## Materials and methods

### Preparation of *Calendula officinalis* extract

Twenty grams of dried flowers of *Calendula officinalis* were collected from Lorestan Agricultural and Natural Resources Research and Education Center and soaked in 120 ml of 50% ethanol or methanol for 72 h in a dark. In the next step, it was centrifuged and passed through the filter and dried at room temperature to yield 8.7% *w*/*w* extract. Then the extract was stored at − 20 °C until further use.

### GC-MS analysis of *Calendula officinalis* extract

Different substances within *Calendula officinalis* extract defined with gas chromatography and mass spectrometry (GC-MS) at the Lorestan University.

### Isolation and culture of MEFs

Isolation and culture of mouse embryonic fibroblasts (MEFs) were performed according to the protocol described elsewhere [12]. In the brief, a pregnant mouse at 13 or 14 days post-coitum (d.p.c) was sacrificed by dislocating of cervical vertebrae and uterine horns were dissected and rinsed in 70% (*v*/*v*) ethanol and placed into a Falcon tube with PBS buffer, without calcium and magnesium ions. The embryos were separated from its embryonic sac and placenta. Then, 1 ml of 0.05% trypsin/EDTA (Gibco, Invitrogen) containing 100 K units of DNase I per each embryo was added and incubated at the room temperature for 15 min. Cells were dissociated by pipetting each 5 min. Trypsin inactivated by the addition of 1 volume of freshly prepared MEF medium. Then, cells were centrifuged and the cell pellet was washed in warm MEF medium.

### MTT assay

To evaluate cell viability, fibroblast cells incubated with different concentrations of *Calendula officinalis* (5 μg/ml, 10 μg/ml, 20 μg/ml, 40 μg/ml, and 50 μg/ml) for 12, 24, 48, and 72 h. Then, 20 μl of MTT (5 mg/ml, Sigma) in PBS solution added into each of the wells, and the plate was further incubated for 4 h. In the next step, 200 μl of DMSO added into each well, incubated for 15 min to dissolve the formed crystal formation and the light absorption measured using an enzyme-linked immunosorbent assay (ELISA) reader. Cell viability expressed as a percentage of absorbance values in treating cells.

### Analysis of TGF β 1 and bFGF gene expression by real-time PCR

MEFs were exposed to different concentrations of ethanolic and methanolic *Calendula officinalis* extracts (5 μg/ml and 10 μg/ml) and then, cells were collected at 12 and 24 h using trypsin/EDTA. Total RNA isolated and the concentration and purity of RNA determined using biophotometer (Eppendorf, Hamburg, Germany). The concentration and quality of the RNA samples confirmed by electrophoresis on 1% denatured agarose gel.

Following procedures were performed:

1. Generation of first strand cDNA with 1 μg total RNA using the cDNA Synthesis Kit (Roche Diagnostics GmbH, Mannheim, Germany).
2. Selection of HPRT as the housekeeping gene.
3. Real-time quantitative PCR by using Rotor-Gene 6000 and SYBR-Green quantitative PCR (qPCR) kit (Jena Bioscience, Cat No. 311S)

Oligonucleotide sequences of the primers and their characteristics are presented in Table 1.

**Table 1:** Sequences of primers for real-time quantitative PCR

Gene	Primer	Product size	Tm
HPRT	Sense:CCTCCTCAGACCGCTTTTT	91	79.5
	Antisense:AACCTGGTTCATCATCGCTAA		
FGF2	Sense: AACGGCGGCTTCTTCCTG	133	78.9
	Antisense:TGGCACACACTCCCTTGATAG		
TGFβ1	Sense: ATTCCTGGCGTTACCTTGG	117	76.9
	Antisense:CCTGTATTCCGTCTCCTTGG		

### Analysis of TGFβ1 and bFGF protein expression by ELISA

MEFs were cultured in 6-well plates (10^5^ cell per well) and were exposed to ethanolic and methanolic extract of *Calendula officinalis* (5 μg/ml, 10 μg/ml) and supernatant were collected at 12 and 24 h. Samples were frozen at − 20 °C. TGF-β1 protein concentration in cell culture supernatants was measured by Ready-Set-GO TGF-β1 cytokine ELISA kit from eBioscience (San Diego, CA, USA, Catalog Number: 88-8350) and FGF ELISA kit (Cat no. ELH-bFGF-001; RayBiotech, Inc., St. Louis, MO).

### Statistical analysis

The statistical analysis of data was done by using SPSS software. Differences between the groups were analyzed by using Wilcoxon test. Differences were considered significant at (*P* < 0.05).

## Results

### GC-MS analysis

GC-MS analysis showed that the main components present in the *Calendula officinalis* extract are carvacrol, thymol, ethyl hexadecanoate, and viridiflorene (Table 2).

**Table 2:** GC-MS analysis showed that the main components present in the *Calendula officinalis* extract are carvacrol, thymol, ethyl hexadecanoate, and viridiflorene

t’R(A)	RI(Calc)	RI(STD)	Similarity	Compound name	Area (%)
4.092	832.5714286	800	83%	Hexanal	1.005961
4.233	846	830	95%	Furfural	1.415797
4.375	859.5238095	851	92%	Furfuryl alcohol	1.453055
4.958	910.5333333	899	80%	Heptanal	1.19225
5.267	931.1333333	998	81%	Octanal	1.254347
5.933	975.5333333	957	94%	Furfural 5-methyl	1.20467
6.175	991.6666667	911	80%	Amyl acetate	3.551913
6.517	1010.432692	950	93%	Glycerin	19.54794
7.592	1062.115385	1112	80%	Heptyl acetate	2.707402
8.092	1086.153846	1106	77%	Maltol	1.800795
10.767	1185.402504	1171	70%	Umbellulone	0.384998
12.2	1230.191458	1228	85%	Citronellol	1.477894
14.433	1295.964654	1285	80%	Bornyl acetate	0.471932
14.642	1301.899736	1290	93%	Thymol	1.328862
14.967	1310.474934	1298	95%	Carvacrol	7.19076
19.658	1432.944162	1439	82%	Aromadendrene	0.422255
20.517	1454.746193	1458	87%	Beta Farnesene	0.981123
22.192	1497.258883	1467	83%	Caryophyllene	0.695479
22.467	1504.249364	1493	93%	Viridiflorene	3.154496
23.433	1528.829517	1524	91%	Delta Cadinene	1.043219
24.708	1561.272265	1549	90%	Elemol	0.34774
26.142	1597.760814	1600	90%	Hexadecane	0.471932
29.125	1674.806202	1658	80%	Eudesmol	2.309985
29.275	1678.682171	1691	80%	Juniper Camphor	0.471932
29.992	1697.209302	1700	87%	Heptadecane	0.397417
39.675	1967.424242	1959	90%	Hexadecanoic acid	0.894188
40.633	1996.454545	1993	93%	Ethyl hexadecanoate	0.558867

### Effect of *Calendula officinalis* extract on MEFs viability

**Fig. 1:**
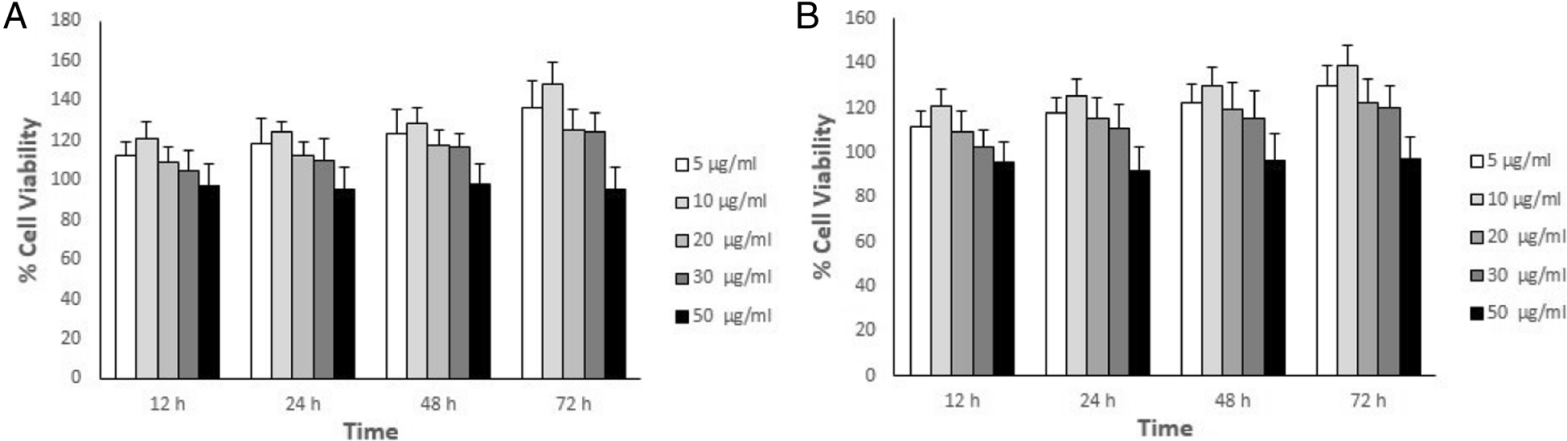
Effect of ethanolic (**a**) and methanolic (**b**) extract of Calendula on MEFs viability. Cells were treated with different concentration of Calendula for 12, 24, 48, and 72 h and cell viability was measured by MTT assay

The results of MTT assay to determine if *Calendula officinalis* ethanolic/methanolic extract affects cell viability of MEFs at different concentration (5 μg/ml, 10 μg/ml, 20 μg/ml, 40 μg/ml) showed that the *Calendula officinalis* extract using both solvents was non-toxic to the cells. The concentrations of 5 μg/ml and10 μg/ml were more suitable for cell proliferation. Therefore, this study was evaluated in these concentrations (Fig. 1).

### TGF β 1 and bFGF gene expression

**Fig. 2:**
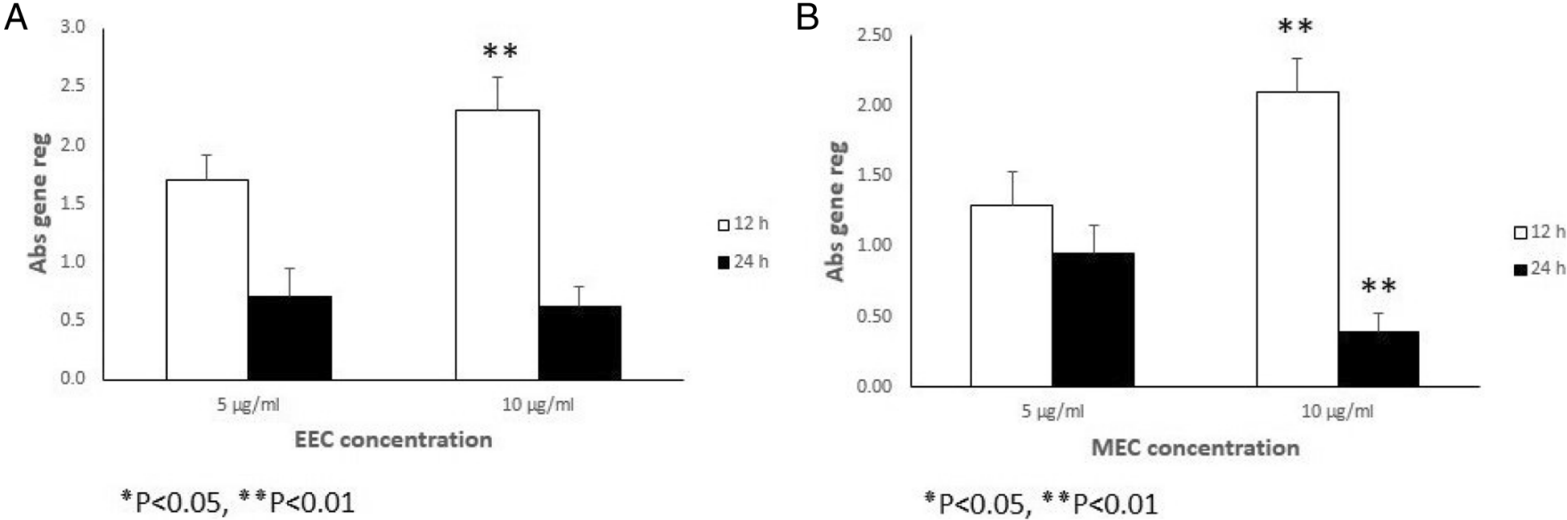
Relative expression of *TGF* β *1* gene in the MEFs that were treated with various concentrations of ethanol (**a**) and methanol (**b**) extracts of Calendula at different time intervals treatment (12 and 24 h). All comparisons were made compared to the control group. *P < 0.05, **P < 0.01. Abs gene reg: Absolute gene regulation

**Fig. 3:**
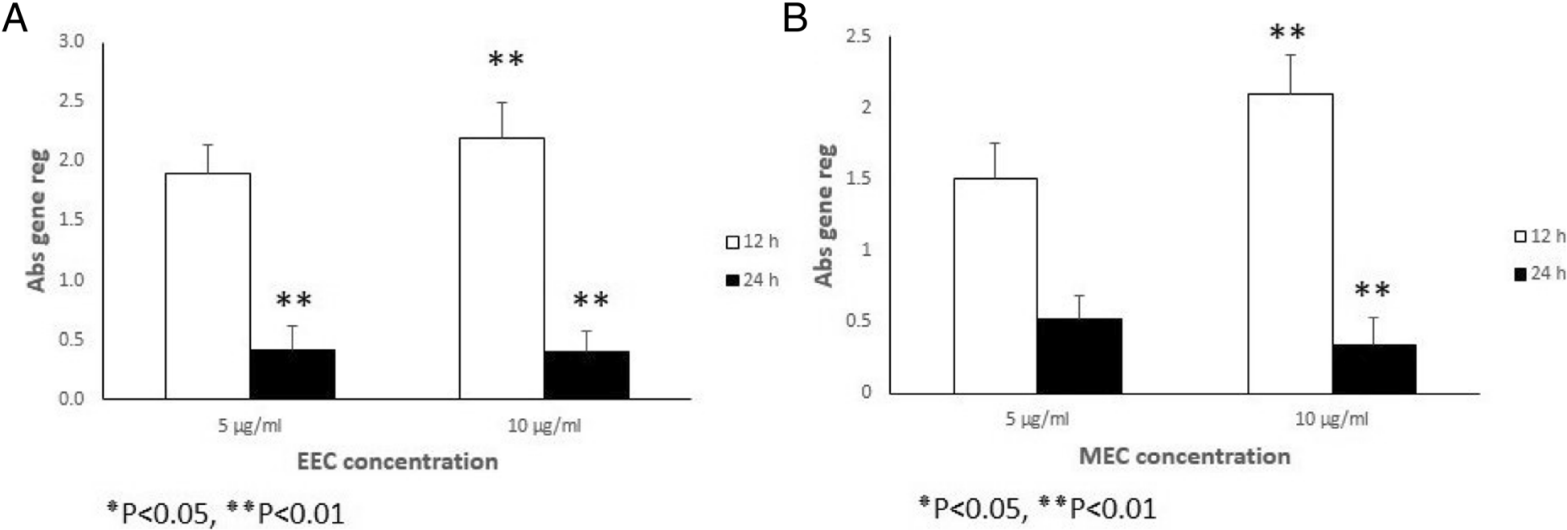
Relative expression of *bFGF* gene in the MEFs that were treated with various concentrations of ethanol (**a**) and methanol (**b**) extracts of Calendula at different time intervals treatment (12 and 24 h). All comparisons were made compared to the control group. ٭*P* < 0.05, ٭٭*P* < 0.01. Abs gene reg: Absolute gene regulation

Analysis of *TGF* β *1* gene expression for the ethanolic extract (Fig. 2a) and the methanolic extract (Fig. 2b), revealed an increase *TGF* β *1* gene expression in the MEFs at different concentrations (5 μg/ml, 10 μg/ml) at 12 h after treatment (*P* < 0.05 and *P* < 0.01 respectively). But the expression of *TGF* β *1* gene remained the same 24 h at the two concentrations after treatment with ethanolic extract (Fig. 2a and Table 3). The methanolic extract results revealed an increase in the expression for the two extracts at 12 h and a decline in the expression of the gene at 24 h (Fig. 2b and Table 4) (*P* < 0.05 and *P* < 0.01 respectively). Gene expression of *bFGF* in the MEFs indicated a similar pattern with *TGF* β *1* expression for both solvent, an increase *bFGF* gene expression in the MEFs at different concentrations (5 μg/ml, 10 μg/ml) at 12 h after treatment (*P* < 0.05 and *P* < 0.01 respectively). But the expression of *bFGF* gene remained the same 24 h at the two concentrations after treatment with ethanolic extract (Fig. 3a and Table 5). The methanolic extract results indicated an increase in the expression for the two extracts at 12 h and a decline in the expression of the gene at 24 h (Fig. 3b and Table 6) (*P* < 0.05 and *P* < 0.01 respectively) (Table 2).

**Table 3:** The total expression ratio of the gene of *TGF*β*1* in the MEFs treated with various concentrations of ethanol extract of Calendula (5 and 10 μg/ml) relative to control group is presented in each time (12 and 24 h after treatment). The statistic test for significance is randomization re-allocation test, implemented in the relative expression software tool. Significant down or upregulations of the genes highlighted

	12 h after treatment		24 h after treatment	
	5 μg/ml	10 μg/ml	5 μg/ml	10 μg/ml
Relative expression	1.7	2.3	0.7	0.62
Standard error	± 0.21	± 0.28	± 0.24	± 0.16
*P* value	0.235	0.001	0.490	0.359
Fold increase/decrease	+ 1.7	+ 2.3	− 1.4	− 1.6

**Table 4:** The total expression ratio of the gene of *TGF*β*1* in the MEFs treated with various concentrations of methanol extract of Calendula (5 and 10 μg/ml) relative to control group is presented in each time (12 and 24 h after treatment). The statistic test for significance is randomization re-allocation test, implemented in the relative expression software tool. Significant down or upregulations of the genes highlighted

	12 h after treatment		24 h after treatment	
	5 μg/ml	10 μg/ml	5 μg/ml	10 μg/ml
Relative expression	1.3	2.1	0.7	0.62
Standard error	± 0.23	± 0.24	± 0.2	± 0.13
*P* value	0.265	0.001	0.661	0.001
Fold increase/decrease	+ 1.3	+ 2.1	− 1.06	− 2.5

**Table 5:** The total expression ratio of the gene of *bFGF* in the MEFs treated with various concentration of ethanol extract of Calendula (5 and 10 μg/ml) relative to control group is presented in each time (12 and 24 h after treatment). The statistic test for significance is randomization re-allocation test, implemented in the relative expression software tool. Significant down or upregulations of the genes highlighted

	12 h after treatment		24 h after treatment	
	5 μg/ml	10 μg/ml	5 μg/ml	10 μg/ml
Relative expression	1.9	2.2	0.42	0.39
Standard error	±0.24	±0.29	±0.19	±0.18
P- value	0.256	0.001	0.001	0.001
Fold increase/decrease	+ 1.9	+ 2.2	− 2.4	−2.5

**Table 6:** The total expression ratio of the gene of *bFGF* in the MEFs treated with various concentration of methanol extract of Calendula (5 and 10 μg/ml) relative to control group is presented in each time (12 and 24 h after treatment). The statistic test for significance is randomization re-allocation test, implemented in the relative expression software tool. Significant down or upregulations of the genes highlighted

	12 h after treatment		24 h after treatment	
	5 μg/ml	10 μg/ml	5 μg/ml	10 μg/ml
Relative expression	1.5	2.1	0.52	0.34
Standard error	± 0.25	± 0.27	± 0.17	± 0.19
*P* value	0.231	0.001	0..275	0.001
Fold increase/decrease	+ 1.5	+ 2.1	− 1.9	− 2.96

### TGFβ1 and bFGF protein expression

**Fig. 4:**
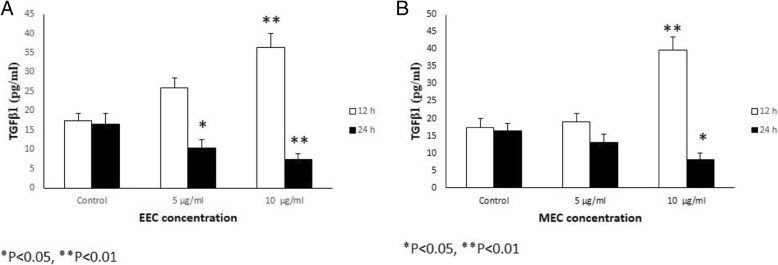
Effect of various concentrations of ethanol (**a**) and methanol (**b**) extracts of Calendula the expression of TGFβ1 protein in the MEFs culture supernatants. The MEFs were treated with different concentration of ethanol and methanol extracts of Calendula (5 and 10 μg/ml) at different time intervals treatment (12 and 24 h) and the expression of TGFβ1 protein was assessed by ELISA

Analysis of TGFβ1 protein expression of the ethanolic extract (Fig. 4a) and the methanolic extract (Fig. 4b) revealed an increase TGFβ1 gene expression in the MEFs at different concentrations (5 μg/ml, 10 μg/ml) at 12 h (*P* < 0.05 and *P* < 0.01 respectively). But the expression of TGFβ1 protein was slightly reduced, 24 h at the two concentrations after treatment with ethanolic extract (Fig. 4a). The methanolic extract results indicated a similar pattern (Fig. 4b) (*P* < 0.05 and *P* < 0.01 respectively).

Protein expression of bFGF in MEFs revealed an increase in bFGF protein expression at different concentrations (5 μg/ml, 10 μg/ml) of 12 h after treatment (*P* < 0.05 and *P* < 0.01 respectively). The same increasing pattern of bFGF protein expression was observed 24 h at the two concentrations after treatment with ethanolic extract. The methanolic extract results indicated a similar pattern with that of the ethanolic extract, as compared with the control (*P* < 0.05 and *P* < 0.01 respectively).

When the ethanolic and methanolic extracts of *Calendula officinalis* were compared, ethanolic extract indicated more effective to stimulate MEFs (Figs. 2, 3, and 4 and Tables 3, 4, 5, and 6).

## Discussion

The results of the present study indicated that both methanol and ethanol extracts of *Calendula officinalis* had non-toxic effects of different concentration. The extracts similar to previous studies increased proliferation of the MEFs [13, 14]. Among different concentrations of Calendula extraction 5 and 10 μg/ml were more suitable for cell proliferation.

Previous investigation revealed that *Calendula officinalis* can inhibit collagen degradation and matrix metalloproteinase (MMP) activity, and induce neovascularization in the chorioallantoic membrane and skin wound.

Quercetin, one of the active components in Calendula, can decrease the expression of tumor necrosis factor-α (TNF-α), interleukin-1β, IL-6, and IL-8 [14–17].

During the wound healing process, fibroblasts play a crucial role. Then, the cells proliferate and migrate into the wound area, synthesis extracellular matrix (EXM), and the express of thick actin bundles as myofibroblasts [18, 19].

Important cytokines that produce by fibroblast are TGFβ1 and bFGF. TGFβ1 and bFGF impacts on cell division, cell migration, cell differentiation, protein expression, and enzyme production and have the potential ability to heal wounds through stimulation of angiogenesis factors and cellular proliferation which affects the ECM production and degradation through their chemotactic role on inflammatory cells and fibroblasts [20].

Several studies indicated that upregulation of TGFβ1 can stimulate the production of fibrotic disease [21, 22].

In the present study, *Calendula officinalis* increased TGFβ1 and bFGF at the first 12 h therefore stimulate wound healing. Then, the decrease of these factors at 24 h suppressed the expression of TGFβ1 and bFGF and may inhibit the fibrotic process. The Calendula at first may upregulate the expression of TGFβ1 and bFGF but after that may downregulate the expression of these genes.

## Conclusion

*Calendula officinalis* not only shows no cytotoxicity effects on MEFs but also stimulates proliferation of these cells. Calendula via increased expression of growth factors (TGFβ1 and bFGF) at the first 12 h, and a decrease of these factors at 24 h after treatment may ameliorate function of fibroblasts in the during wound healing.

## Abbreviations

bFGF: Basic fibroblast growth factor
cDNA: Complementary deoxyribonucleic acid
DMEM: Dulbecco’s modified Eagle’s medium
ELISA: Enzyme-linked immunosorbent assay
FBS: Fetal bovine serum
HPRT: Hypoxanthine phosphoribosyltransferase
MEF: Mouse embryonic fibroblast
MTT: 3-(4, 5-Dimethylthiazol-2-yl)-2, 5-diphenyltetrazolium bromide
nm: Nanometer
PBS: Phosphate-buffered saline
Pg/ml: Picograms per milliliter
Real-time PCR: Real-time polymerase chain reaction
TGFβ1: Transforming growth factor β1
Tm: Melting temperature
TMB: 3, 3′, 5, 5′-Tetramethylbenzidine
